# Tobacco use and associated mental symptoms and health risk behaviours amongst individuals 15 years or older in South Africa

**DOI:** 10.4102/sajpsychiatry.v26.i0.1499

**Published:** 2020-11-09

**Authors:** Karl Peltzer, Supa Pengpid

**Affiliations:** 1 Department of Research Administration and Development, University of Limpopo, Turfloop, South Africa; 2 ASEAN Institute for Health Development, Mahidol University, Salaya, Phutthamonthon, Nakhon Pathom, Thailand

**Keywords:** mental symptoms, tobacco use, health risk behaviour, post-traumatic stress disorder, South Africa

## Abstract

**Background:**

Tobacco use may deteriorate mental health and increase health risk behaviours.

**Aim:**

The aim of this investigation was to identify associations between tobacco use and mental illness symptoms and health risk behaviours in individuals 15 years or older in South Africa.

**Setting:**

Community-based national population sample in South Africa.

**Methods:**

Cross-sectional data were analysed from the ‘South African National Health and Nutrition Examination Survey (SANHANES-1) 2012’, using a sample of 15 310 individuals 15 years or older (median age 33 years). Measures included information on tobacco use, sociodemographic factors, mental symptoms and health risk behaviour.

**Results:**

Compared to non-tobacco users, daily tobacco users were associated with psychological distress and post-traumatic stress disorder (PTSD) in adjusted logistic regression analysis, and with sleeping problems in unadjusted analysis. Past tobacco use, less than daily, and daily tobacco use were highly associated with a drinking problem. In terms of dietary variables, less than daily and daily tobacco use increased the odds of inadequate fruit intake and salty food intake, and daily tobacco use decreased the odds of fast food consumption. Past tobacco use, less than daily, and daily tobacco use were inversely associated with physical inactivity, and daily tobacco use was associated with not always washing hands before eating.

**Conclusions:**

The study showed that compared to non-tobacco users, daily tobacco users had significantly poorer mental health (psychological distress and PTSD) and increased odds for several health risk behaviours (drinking problem, inadequate fruit intake, salty food consumption and not always washing hands before eating) as compared to non-tobacco users.

## Introduction

‘Tobacco kills more than 8 million people each year. Around 80% of the world’s 1.1 billion smokers live in low- and middle-income countries.’^1^ Smoking is known to cause various illnesses, including ‘cancer, heart disease, stroke, lung diseases, diabetes, and chronic obstructive pulmonary disease (COPD)’.^2^ Fewer studies have linked tobacco use with mental morbidity and health-compromising behaviours.

Some studies found a probable relationship between tobacco use and mental illness, including anxiety and depression in adults.^3,4,5,6,7^ In a systematic review, Kearns et al.^8^ found that:

Smokers were approximately twice more likely to have posttraumatic stress disorder (PTSD) than non-smokers in the general population, and individuals with PTSD were approximately twice as likely to be current smokers. (p. 1056)

In another systematic review ‘high rates of comorbidity of PTSD and tobacco use’ were found.^9^ Several cross-sectional studies showed that the prevalence of psychological distress was significantly higher in tobacco users or smokers compared to non-tobacco users or non-smokers.^10,11,12,13^ Few investigations identified an association between tobacco use and sleep disorder,^14^ poor sleep quality^15^ and short sleep.^16^ Many studies found an association between tobacco use and alcohol consumption,^7,17,18,19,20,21^ hazardous alcohol use^15,22^ and illicit drug use.^23^

A number of studies found that compared to non-smokers, smokers had higher odds of having unhealthy nutritional status and dietary behaviour, such as poor diet quality,^22,24^ less compliance with dietary recommendations,^24^ fast-food consumption,^21,25^ more high-fat foods intake,^26,27^ consumption of foods high in sugar and soft drinks,^17,21,25,26,28^ fewer fruit and/or vegetables intake,^17,19,20,21,26,27,28^ high sodium consumption^15,19,26^ and less likely to eat milk and dairy products.^21,28^ In addition, tobacco use was associated with physical inactivity,^22,29^ more exercise,^28^ sedentary behaviour^29^ and poor oral hygiene.^15^

Individuals’ habitual participation in a single health-risk behaviour substantially contributes to morbidity and mortality (e.g., tobacco use, daily fast food intake, etc.); however, more concerning is the impact of typically co-occurring or clustering of multiple health-risk behaviours.^30^ (p. 194)

How and if tobacco use is associated with mental illness symptoms and health-compromising behaviours will provide a better understanding on potential relationships across risk behaviours.^30^ This study aimed to identify associations between tobacco use, mental illness symptoms and health risk behaviours in individuals 15 years or older in South Africa.

## Method

### Sample and procedure

The ‘South African National Health and Nutrition Examination Survey (SANHANES-1) 2012’ is a nationally cross-sectional representative survey of the ‘non-institutionalized population of South Africa’ to measure the nutrition and health status of the population.^31^ This survey employed a ‘multi-stage disproportionate stratified cluster sampling design’.^31^ In all:

500 enumeration areas (EAs) representative of the socio-demographic profile (stratified by province, locality type and race) of South Africa were identified, and a random sample of 20 households was selected from each. EA.^31^ (p. 2)

‘All persons residing in the selected households were eligible to participate.’^31^ The current analysis was restricted to persons 15 years and older. Data for this analysis were collected by administering questionnaires to participants (conducting face-to-face interviews).^31^ ‘Interviews were conducted by trained fieldworkers.’^31^ The questionnaire was ‘translated from English to all national languages and then back translated and pre-tested prior to the main survey’.^31^ The individual interview response rate of participants was 92.6%.^31^

Sample size estimation:

Guided by the requirement of an acceptable precision of estimates per reporting prevalence of a health variable, and the requirement for measuring change over time, the total sample size of 10 000 households was based on the minimum sample sizes needed for each reporting health variable, and also took into account the multistage cluster sampling design and the expected response rates. Under the assumption that 75% of the 10 000 households in the sampling frame would agree to participate, the survey would yield 7500 valid contactable households with eligible survey participants.^31^ (p. 45)

### Measures

#### Outcome variables

The outcome variables included mental symptoms, substance use and health risk behaviour indicators.

#### Mental symptoms and substance use indicators

*Psychological distress* was measured with the 10-item Kessler scale.^32^ ‘This scale inquires about psychological distress symptoms experienced in the past 30 days, with response options ranging from 1 = never to 5 = all of the time.’^32^ ‘Scores of all items are summed, with scores 20 or more indicating mild, moderate or severe psychological distress.’^32^ The Kessler 10 scale has been ‘validated for use in the South African context’^33^ (Cronbach’s alpha 0.93).

*Post-traumatic stress disorder* was measured with the ‘17-item Davidson Trauma Scale (DTS)’ that assesses ‘all primary DSM-IV symptoms of PTSD related to intrusion, avoidance and hyperarousal symptoms’.^34^ Participants had preliminary PTSD ‘if they score at least one re-experiencing, three avoidance/numbing and two hyperarousal phenomena at a frequency of at least twice in the previous week’^34^ (Cronbach’s alpha 0.94). The DTS has not been validated for the South African context.^35^

*Sleeping problems* were measured with one question on the ‘severity of nocturnal sleep problems, such as falling asleep, waking up frequently during the night, or waking up too early in the morning’, and one question on the ‘severity of difficulty with daytime functioning, not feeling rested and refreshed during the day, for example, feeling tired, not having energy’; responses ranged from ‘0 = none to 4 = extreme/cannot do’ (Cronbach’s alpha 0.82). Sleeping problems were defined as having 4–8 scores.^36^

Drinking problem or hazardous alcohol use was assessed with the ‘Alcohol Use Disorders Identification Test–Consumption (AUDIT-C)’.^37^

The AUDIT-C has 3 questions and is scored on a scale of 0–12. Each AUDIT-C question has 5 answer choices valued from 0 points to 4 points. In men, a score of 4 or more is considered positive, optimal for identifying hazardous drinking or active alcohol use disorders. In women, a score of 3 or more is considered positive.^37^ (p. 1091)

(Cronbach’s alpha 0.89). The AUDIT-C is a shorter version of the AUDIT-10, which has been validated in South Africa.^38,39^

#### Health risk behaviour indicators

*Fruit consumption*: ‘How many fruits do you usually eat per day?’ *Vegetable consumption*: ‘How many portions of vegetables, excluding potatoes, do you usually eat per day?’^31^

*Other dietary items* included the consumption of ‘Food from fast food outlets (takeaways, for example, pizza, chicken, fish, etc.)?’ ‘Processed meat, e.g., sausages, polony, cold cuts, Vienna’s, Fankfurters, Russians, salami?’ ‘Snacks, such as chips, crisps, *mazimba*, etc.?’ and ‘Sweetened cold drink (gas/fizzy cold drink and reconstituted)?’ Responses were classified as 4–6 times in the past week.^31^ In addition, participants were asked, ‘Do you prefer to eat your food usually very salty, lightly salted or not salted?’^31^

*Physical activity* was measured with the ‘General Physical Activity Questionnaire (GPAQ)’,^38,39,40^ and grouped into ‘low, moderate, and high physical activity’ according to GPAQ guidelines.^40^ The GPAQ has been previously validated in nine countries, including in South Africa, and found an acceptable measure of physical activity.^41^

*Sedentary behaviour* was sourced from the ‘time spend sitting or reclining (lying) on a usual weekday or weekend day (excluding sleeping)’,^42^ and defined as eight or more hours per day.^43^

*Not always washing hands* before eating was sourced from the question ‘How often do you wash your hands before eating?’^31^

#### Exposure variable

Tobacco use was measured with questions on the ‘history of tobacco smoking and use of other tobacco products, duration and frequency of use’, as follows:

Do you currently smoke tobacco? (Yes, daily; Yes, less than daily; No, not at all) Do you currently use other tobacco products, such as hand-rolled cigarettes, pipes full of tobacco, cigars, cheroots, cigarillos, hookah, hubbly bubbly, water pipe, electronic cigarettes, snuff, chewing tobacco, smokeless tobacco? (Yes, daily; Yes, less than daily; No, not at all).^31^ (p. 9)

Past tobacco use:

In the past, did you smoke Hand-rolled cigarettes; Pipes full of tobacco; Cigars, cheroots, or cigarillos; Hookah, hubbly bubbly or water pipe sessions; Electronic cigarettes; Any other, specify. In the past, have you used snuff? …chewing tobacco? …other smokeless tobacco?^31^ (p. 9)

Based on these questions, participants were divided into never, past, less than daily and daily tobacco users.

#### Confounding variables

*Demographic data* included employment status, population group, sex, age and residence status.^31^

*Self-rated health status* was assessed with the question: ‘In general, how would you rate your health today?’^31^

*Functional disability* was assessed with the 12-item ‘WHO Disability Assessment Schedule, version 2.0 (WHODAS-II)’.^44^ (Cronbach’s alpha 0.90) ‘The WHODAS-II score was transformed into a score of 0 to 100, with 25% or more indicating moderate to extreme functional disability’.^44^

*Chronic conditions* were assessed as follows:

Has a doctor or nurse or health worker at a clinic or hospital told you that you have had any of the following conditions? High blood pressure, stroke, heart disease, heart attack or angina (chest pains), high blood cholesterol, high blood sugar or sugar diabetes.^31^ (p. 381)

#### Data analysis

Frequency, median and interquartile range were calculated to describe the sample and its indicators. Unadjusted and adjusted logistic regression using Taylor series linearisation was used to determine the associations between tobacco use categories (never, past, less than daily and daily use) and health outcome variables (mental symptoms and health-compromising behaviours). The final model was adjusted for relevant confounders, age, sex, population group, employment status, residence status, self-rated health status, chronic conditions and functional disability. The independent variable found to have significant associations with a health outcome variable in the univariate model was used in the multivariable logistic regression model. Missing data were excluded from the calculations. *P* < 0.5 was accepted as significant. ‘STATA 15.00 (StatCorp LP, College Station, TX)’ was used for all statistical analyses, taking into account the weights and clustering effects of the complex sample design.

### Ethical consideration

Informed written consent was obtained from participants. The study protocol was approved by the research ethics committee (REC) of the Human Sciences Research Council (REC 6/16/11/11).

## Results

The sample included 15 310 individuals that were 15 years or older (median age of 33 years, interquartile range: 23–47 years), of which 54.3% were female, 78.4% were black African by population group, 63.3% were employed, 63.4% lived in urban areas, 22.4% had a moderate, bad or very bad self-rated health status, 22.8% had one or more chronic conditions and 9.2% had a functional disability. Almost one in five participants (17.2%) screened positive for psychological distress, 2.1% for PTSD, 7.1% for sleeping problems and 20.3% for drinking problem. More than two in five persons (42.3%) had fruits less than once in a day, 42.0% had vegetables less than once in a day, 11.0% had processed meat frequently (≥ 4 times per week), 15.4% had snacks frequently (≥ 4 times per week), 10.6% consumed soft drinks daily, 46.0% had fast food once or more times in a week and 7.0% preferred to usually eat their food very salty. Almost half (47.0%) of the study population engaged in low physical activity, 13.4% in sedentary behaviour (≥ 8 hours per day), and 18.2% did not always wash hands before eating. In all, 77.4% of participants had never used tobacco, 3.8% used tobacco in the past, 2.5% not daily and 16.2% used tobacco daily.

### Associations with mental symptoms and health-compromising behaviour

Compared to non-tobacco users, daily tobacco users were associated with psychological distress and PTSD in adjusted logistic regression analysis, and with sleeping problems in unadjusted analysis. Past tobacco use, less than daily, and daily tobacco use were highly associated with drinking problem. In terms of dietary variables, less than daily and daily tobacco use increased the odds of inadequate fruit intake, and daily tobacco use decreased the odds of fast food consumption. Past tobacco use, less than daily, and daily tobacco use were inversely associated with physical inactivity, and daily tobacco use was associated with not always washing hands before eating (see Table 1).

**TABLE 1:** Associations between tobacco use categories and outcome variables.

Outcome variables	Tobacco use	UOR	95% CI	AOR	95% CI ^a^
Psychological distress (17.2%)	Never	1	Reference	1	Reference
	past	1.61	1.20, 2.16**	1.23	0.83, 1.84
	< daily	1.10	0.79, 1.53	0.96	0.66, 1.40
	daily	1.25	1.07, 1.47**	1.49	1.22, 1.83***
Post-traumatic stress disorder (2.1%)	Never	1	Reference	1	Reference
	past	1.84	1.11, 3.03*	1.28	0.72, 2.28
	< daily	2.36	1.27, 4.38**	1.82	0.79, 4.19
	daily	1.83	1.29, 2.58***	1.83	1.23, 2.71**
Sleeping problems (7.1%)	Never	1	Reference	1	Reference
	past	1.93	1.34, 2.78***	1.09	0.68, 1.51
	< daily	1.40	0.91, 2.17	1.15	0.60, 2.19
	daily	1.27	1.00, 1.60*	1.32	0.95, 1.83
Problem drinking (20.3%)	Never	1	Reference	1	Reference
	past	2.29	1.69, 3.20***	2.26	1.58, 3.23***
	< daily	3.94	2.83, 5.48***	3.23	2.23, 4.68***
	daily	7.15	6.23, 8.22***	6.00	5.09, 7.07***
Fruits (< 1 per day) (42.3%)	Never	1	Reference	1	Reference
	past	1.21	0.89, 1.65	1.37	0.96, 1.95
	< daily	1.49	1.13, 1.97**	1.44	1.04, 2.00*
	daily	1.34	1.18, 1.53***	1.33	1.15, 1.54***
Vegetables (< 1 per day) (42.0%)	Never	1	Reference	-	-
	past	1.14	0.86, 1.52	-	-
	< daily	1.14	0.86, 1.52	-	-
	daily	1.06	0.92, 1.21	-	-
Processed meat (≥ 4 times per week) (11.0%)	Never	1	Reference	1	Reference
	past	0.55	0.33, 0.90*	0.60	0.34, 1.08
	< daily	0.76	0.45, 1.29	0.77	0.41, 1.44
	daily	0.75	0.60, 0.94*	0.80	0.62, 1.02
Snacks (≥ 4 times per week) (15.4%)	Never	1	Reference	1	Reference
	past	0.88	0.63, 1.23	0.88	0.55, 1.41
	< daily	0.88	0.59, 1.31	0.99	0.65, 1.51
	daily	0.75	0.61, 0.92**	0.94	0.73, 1.19
Soft drink (daily) (10.6%)	Never	1	Reference	-	-
	past	1.23	0.64, 2.35	-	-
	< daily	1.14	0.74, 1.75	-	-
	daily	0.78	0.59, 1.04	-	-
Fast food (≥ 1 week) (46.0%)	Never	1	Reference	1	Reference
	past	0.82	0.60, 1.12	0.89	0.56, 1.40
	< daily	0.81	0.59, 1.10	0.85	0.62, 1.17
	daily	0.71	0.61, 0.83***	0.68	0.57, 0.81***
Food very salty (7.0%)	Never	1	Reference	1	Reference
	past	2.00	1.39, 2.86***	1.95	1.33, 2.85***
	< daily	2.29	1.44, 3.65***	1.84	1.23, 2.77**
	daily	1.85	1.49, 2.29***	1.74	1.38, 2.18***
Physical activity (low) (47.0%)	Never	1	Reference	1	Reference
	past	0.66	0.48, 0.90***	0.59	0.39, 0.90*
	< daily	0.49	0.35, 0.69***	0.60	0.42, 0.85**
	daily	0.64	0.54, 0.76***	0.63	0.52, 0.76***
Sedentary (8 or more hours per day) (13.4%)	Never	1	Reference	-	-
	past	0.74	0.45, 1.22	-	-
	< daily	0.70	0.44, 1.11	-	-
	daily	0.99	0.83, 1.18	-	-
Wash hands before eating (not always) (18.2%)	Never	1	Reference	1	Reference
	past	1.25	0.83, 1.68	1.08	0.78, 1.51
	< daily	1.39	1.02, 1.90*	0.92	0.64, 1.34
	daily	1.31	1.10, 1.55**	1.30	1.07, 1.57**

UOR, Unadjusted Odds Ratio; AOR, Adjusted Odds Ratio.

a , Adjusted for age, sex, population group, employment status, residence status, self-rated health status, chronic conditions and functional disability.

*** , *p* < 0.001;

** , *p* < 0.01;

* , *p* < 0.05.

## Discussion

In this large cross-sectional nationally representative study of persons 15 years or older in South Africa, compared to non-tobacco users, daily tobacco users had significantly poorer mental health (psychological distress and PTSD) and increased odds for several health-compromising behaviours (drinking problem, inadequate fruit intake, intake of salty food and not always washing hands before eating) as compared to non-tobacco users in adjusted analysis. These findings are largely consistent with previous research.^3,4,5,6,7,10,11,12,15,17,18,19,20,25,26,27^ It is possible that compared to non-tobacco users, ‘tobacco users engage to a greater extent in risk denial not only with tobacco use but also with other health risk behaviours’.^15,26^

A strong dose-response relationship was found between tobacco use and drinking problem in this study. The high co-occurrence between tobacco and alcohol use is likely to increase with higher rates of consumption of each substance.^45,46,47^ Possible reasons for this heightened co-occurrence risk between tobacco and alcohol use include behavioural, pharmacological and genetic factors.^46,47,48^ The combined use of tobacco and alcohol use is particularly harmful causing increased mortality,^49^ emphasising the need to target both substances in intervention efforts.^46,47^ A dose-response relationship of tobacco use (from never, past, < daily to daily) was in this study identified in other four health indicators (psychological distress, PTSD, poor diet, and poor general hygiene). Similar results were found in a study amongst adolescents^21^ and university students.^15^

Some studies^14^ identified an association between tobacco use and sleep disorder, whilst in this study, this was found in unadjusted analysis. Most studies^22,29^ found an association between tobacco use and physical inactivity and sedentary behaviour, whilst this study found a negative association with physical inactivity and no association with sedentary behaviour. In a study amongst adolescents, tobacco use was also associated with more exercise,^28^ and amongst adults in Tunisia, no significant differences were found between male smokers and non-smokers in terms of physical inactivity.^26^

Previous studies^21,25^ also found associations between tobacco use and other unhealthy dietary patterns, such as fast-food and soft drink consumption, whilst this study found an inverse association between daily tobacco use and fast-food consumption and no association with soft drink consumption. It is possible that the higher cost of daily tobacco use reduces cash availability to buy fast food. Mathew et al.^50^ have proposed a model for persistent smoking amongst depressive smokers:

[*E*]xperiencing greater increases in the expected value of smoking in the face of three motivational states (low positive affect, high negative affect and cognitive impairment), which promotes goal-directed choice of smoking behaviour over alternative actions. (p. 401)

Similar mechanisms may be at play with other mental problems, such as anxiety, and possibly health risk behaviours.

The findings of the study support the importance of understanding differences in mental illness symptoms (psychological distress, PTSD symptoms, sleeping problems and drinking problem) and health risk behaviours (such as poor diet, physical inactivity and poor general hygiene behaviour) by tobacco use status for health promoters assisting the general population in making healthy behaviour choices. In addition to tobacco use cessation, tobacco users should be educated about their mental illness symptoms and other health risk behaviours, and that health promotion on healthy diet, well-being, sleep hygiene and other health risk behaviours, such as general hygiene behaviour, should be integrated into tobacco use prevention and control strategies in the general population in South Africa.^29^

## The strength and limitations of the study

The strength of the study was the utilisation of a nationally representative sample of people 15 years and older in South Africa. The major limitation of this study was that it was cross-sectional. Therefore, we were not able to determine the direction of the relationships between tobacco use and mental illness symptoms and health risk behaviours. Further, the measures used were by self-report which may have biased responses.

## Conclusion

In this large cross-sectional national study in South Africa, compared to non-tobacco users, daily tobacco users had significantly poorer mental health (psychological distress and PTSD) and increased odds for several health-compromising behaviours (drinking problem, inadequate fruit intake, salty food consumption and not always washing hands before eating) as compared to non-tobacco users in adjusted analysis. The found associations may be taken into account in health promotion activities targeting the reduction of tobacco use.
